# The adaptive buffered force QM/MM method in the CP2K and AMBER software packages

**DOI:** 10.1002/jcc.23839

**Published:** 2015-02-03

**Authors:** Letif Mones, Andrew Jones, Andreas W Götz, Teodoro Laino, Ross C Walker, Ben Leimkuhler, Gábor Csányi, Noam Bernstein

**Affiliations:** [a] Engineering Department, University of CambridgeCambridge, CB2 1PZ, United Kingdom; [b] Institute for Condensed Matter and Complex Systems, School of Physics and Astronomy, University of EdinburghEdinburgh, EH9 3JZ, United Kingdom; [c] San Diego Supercomputer Center, University of California San DiegoLa Jolla, California, 92093; [d] Mathematical and Computational Sciences Department, IBM Research–ZurichSäumerstrasse 4, 8803, Rüschlikon, Switzerland; [e] Department of Chemistry and Biochemistry, University of California San DiegoLa Jolla, California, 92093; [f] The Maxwell Institute and School of Mathematics, University of EdinburghEdinburgh, EH9 3JZ, United Kingdom; [g] Center for Computational Material Science, Naval Research LaboratoryWashington, DC, 20375

**Keywords:** quantum-mechanics/molecular-mechanics, adaptive quantum-mechanics/molecular-mechanics, force-mixing, multiscale

## Abstract

The implementation and validation of the adaptive buffered force (AdBF) quantum-mechanics/molecular-mechanics (QM/MM) method in two popular packages, CP2K and AMBER are presented. The implementations build on the existing QM/MM functionality in each code, extending it to allow for redefinition of the QM and MM regions during the simulation and reducing QM-MM interface errors by discarding forces near the boundary according to the buffered force-mixing approach. New adaptive thermostats, needed by force-mixing methods, are also implemented. Different variants of the method are benchmarked by simulating the structure of bulk water, water autoprotolysis in the presence of zinc and dimethyl-phosphate hydrolysis using various semiempirical Hamiltonians and density functional theory as the QM model. It is shown that with suitable parameters, based on force convergence tests, the AdBF QM/MM scheme can provide an accurate approximation of the structure in the dynamical QM region matching the corresponding fully QM simulations, as well as reproducing the correct energetics in all cases. Adaptive unbuffered force-mixing and adaptive conventional QM/MM methods also provide reasonable results for some systems, but are more likely to suffer from instabilities and inaccuracies. © 2015 The Authors. Journal of Computational Chemistry Published by Wiley Periodicals, Inc.

## Introduction

In a quantum-mechanics/molecular-mechanics (QM/MM) simulation,[1] an atomistic system is described using a QM model of bonding in a small, spatially localized region, while the remainder of the system is described with a MM model. The QM description makes it possible to describe processes that the typically nonreactive MM model cannot, such as changes of charge state or covalent bond rearrangement. The MM description of the rest of the system provides the appropriate far field structure and mechanical and/or electrostatic boundary conditions for the QM description. The two descriptions can interact directly through covalent, electrostatic, or other nonbonded interactions, and indirectly through the structure of the MM system. Capturing such long range interactions can be essential even for the description of the local structure: in an enzyme the reaction involves residues that are kept in place by the structure of the rest of the protein; in some cases long range electrostatic effects play a direct role in the reaction.[2,3] QM/MM methods have matured over the past few decades into an essential tool for modeling chemical reactions of complex systems.

For a QM/MM method to describe the complete system accurately, the individual methods used for the QM and MM descriptions must be appropriate for the configurations and processes in their respective regions, and the interaction between them must be accounted for. The dominant approach, which we will call conventional QM/MM (Conv-QM/MM) here, is to fix the set of atoms in the QM and MM subsystems and define the total energy of the system as a sum of the QM energy of the QM region, the MM energy of the MM region, and an interaction energy. The interaction term can include the nonbonded and electrostatic energies of MM descriptions of the QM atoms in the field of the MM atoms (“mechanical embedding”),[4] or it may include the effect of the MM electrostatic field on the QM description, including the explicitly described electron density (“electrostatic embedding”).[4] If covalent bonds across the QM-MM interface are present, they must be capped in some way so as to eliminate dangling bonds in the QM subsystem, for example, using H atoms,[5] generalized hybrid orbitals[6] or pseudopotentials.[7] It is difficult to devise a general algorithm for this task that works satisfactorily for all bonding topologies that are likely to be encountered. The accuracy of the conventional approach depends on the appropriateness of using a fixed set of atoms in the QM region, and on the ability of the QM-MM interaction term to eliminate the fictitious boundary effects in the QM and MM subsystem calculations.

Carrying out QM/MM simulations on different sized QM regions shows that widely used interaction terms lead to significant errors in the atomic forces near the QM-MM interface when compared to calculations using very large QM regions or which describe the entire system quantum mechanically using periodic boundary conditions (we will refer to the latter as “fully QM”).[8]–[11] Although in many cases the effect on relevant observables can be small, these errors can be very problematic when the set of QM atoms is allowed to change. In such adaptive methods,[12]–[19] which are used to enable the QM region to move or species to diffuse in or out of the reaction site, errors near the interface can lead to an instability and a net flux of atoms between the QM and MM regions resulting in unphysical density variations.[20,21]

There are a number of fundamental issues that must be addressed in the design of any method that couples different descriptions in different regions of a single system. The way they are addressed can have particular implications for adaptive simulations, which may be different from the way the choices affect simulations where the set of atoms in each subsystem is fixed. One choice is whether the coupling is formulated in terms of energy[13,15,16,18,19,22] or forces.[12,14,17,20,21,23]–[25] If it is formulated in terms of energy, the total energy of the coupled system can be defined, and changes of that energy as atoms or molecules switch between descriptions can adversely affect the simulation. This can be represented as a difference in chemical potential of the switching species being described with the two models. A mismatch at any point in space for any molecular conformation will lead to unphysical forces on atoms as they switch description, leading to transport of atoms to the lower chemical potential region. Coupling in terms of forces can avoid this chemical potential mismatch effect, at the cost of forgoing energy conservation because no total energy can be defined, due to the nonconservative nature of the forces used to drive the dynamics. This tradeoff motivated the choice to use a force-based approach in our work, as well as in the Hot Spot[12] and difference-based adaptive solvation (DAS)[17] methods. The use of nonconservative forces would lead to unstable molecular dynamics trajectories, which we avoid using adaptive thermostats. These have been shown to sample the correct distribution even in the presence of net heat generation.[26]

Another choice is whether the transition between the two descriptions is abrupt or continuous. An abrupt transition leads to discontinuities in the dynamics as atoms suddenly switch from one region to another. Using a transition region can make the energy or forces continuous by smoothly interpolating between multiple calculations, but increases the number of force calculations that must be performed. While many published methods use transition regions to smooth out such switching discontinuities,[12]–[19,27] we have found that using abrupt transitions within a force-mixing approach does not seem to significantly affect the accuracy of average structures and free energy profiles.[20,21,28]

The third choice is how the errors near the interface between the two regions are handled. Energy based methods are formulated in terms of an MM energy, a QM energy, and the interaction term, and the accuracy of the last one determines this error. Adaptive methods like Our Own N-layered Integrated molecular Orbital and Molecular mechanics eXchange of Solvent (ONIOM-XS)[13] and Sorted adaptive partitioning (SAP)[15,16] simply combine a weighted sum of several such calculations, and therefore include a weighted sum of interface related errors. Methods that mix a quantity that can be localized to each atom can, in general, improve on this using buffer, as we explain below. Because the energy, especially in the QM description, can not be localized to each atom, such mixing is generally applied to forces.[12,14,17,19] The buffer regions used to improve boundary force errors are conceptually distinct from the transition regions mentioned above that help smooth discontinuities.

Over the past few years we have developed the adaptive buffered force-QM/MM method (AdBF-QM/MM), which uses force-mixing, abrupt transitions, and buffers to reduce the effect of interface errors and enable stable adaptive simulations.[20] Many other published methods can also be characterized in terms of the above choices, and we summarize these in Table1. The ABRUPT method[19] is equivalent to a Conv-QM/MM simulation where atoms are allowed to switch abruptly between the two descriptions without buffers. The Hot Spot method[12] uses force-mixing with transitions that are interpolated over a region of about 0.5 Å, but no buffers. SAP,[15] ONIOM-XS,[13] and DAS[17] all use smooth transitions and no buffers, but the first two use an energy based coupling while the last uses force-mixing. The SAP and DAS methods require one calculation per molecule in the transition region, and the ONIOM-XS method is limited to a single molecule in that region.

**Table 1:** Important features of conventional and adaptive QM/MM methods, including the four methods used here as well as related previously published methods.

Method	Adaptive?	Mixed quantity	Abrupt transition?	Number of QM calculations/step	Buffer?	Related method
Conventional QM/MM (Conv-QM/MM)	no	energy	yes	1	no	
Adaptive Conventional QM/MM (AdConv-QM/MM)	yes	energy	yes	1	no	ABRUPT[19]
Adaptive Unbuffered Force-mixing QM/MM (AdUF-QM/MM)	yes	force	yes	2	no	Hot Spot[12]
Adaptive Buffered Force-mixing QM/MM (AdBF-QM/MM)	yes	force	yes	2	yes	
Difference-based Adaptive Solvation (DAS)[17]	yes	force	no	*N*	no	
Sorted Adaptive Partitioning (SAP)[15]	yes	energy	no	*N*	no	
“Our Own N-layered Integrated molecular Orbital and Molecular mechanics eXchange of Solvent”						
ONIOM-XS[13]	yes	energy	no	*N* = 2	no	

*N* is the number of atoms or molecules in the transition region. Note that “Our Own” in the full name of the ONIOM-XS method is simply part of the name, and does not indicate that it is the work of the authors of this article.

In previous publications we tested the AdBF-QM/MM method on the structure of bulk water,[20] as well as the free energy profiles of two reactions in water, nucleophilic substitution in methyl chloride and the deprotonation of tyrosine.[21] Here we describe the new implementation of the AdBF-QM/MM method in two popular software packages, CP2K[29] and AMBER.[30,31] The implementations extend the QM/MM capabilities of the packages, and with appropriate choice of parameters can be used to carry out adaptive QM/MM simulation with or without buffering and force-mixing.

We test the different variants using a variety of QM models, including density functional theory (DFT) and semiempirical (SE) quantum mechanical models. We validate the implementations by repeating the earlier test of the structure of bulk water[20] using additional QM models, and present the results of two new tests, the free energy profiles of dimethyl-phosphate hydrolysis and the autoprotolysis of water in the presence of a zinc ion. These biologically relevant and widely studied reactions were chosen as challenging tests due to the significant charge transfer that leads to strong interactions between the reactants and nearby solvent molecules.

## Methodology

### Overview of adaptive buffered force-QM/MM method

**Figure 1:**
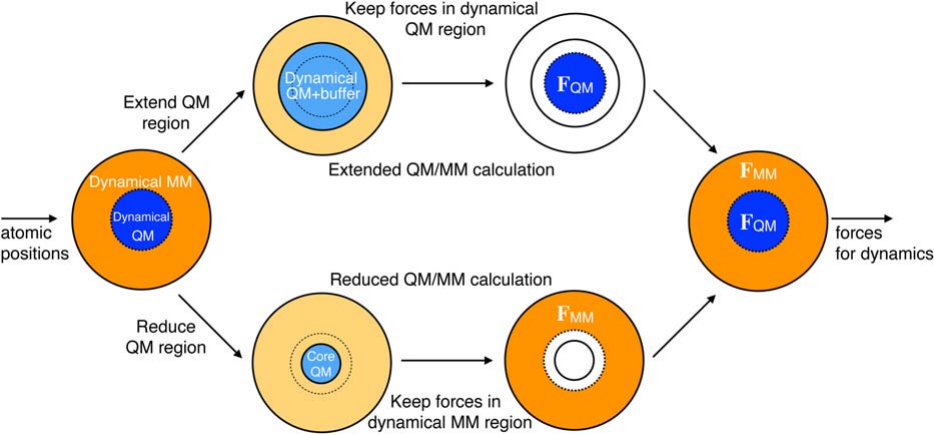
Flowchart of the AdBF-QM/MM method. First column: divide system into dynamical QM (blue) and dynamical MM (orange) regions. Second column: set up two QM/MM calculations, extended where the QM region is enlarged by a buffer region (top), and reduced where the QM region is shrunk as much as possible, perhaps to nothing (bottom). Third column: select forces from each of the two calculations, keeping QM forces in dynamical QM region (blue) from extended QM/MM calculation (top) and keeping MM forces in dynamical MM region (orange) from reduced QM/MM calculation (bottom). Fourth column: combine forces from two calculations into complete set for dynamics.

In the AdBF-QM/MM method the atomic forces that are used in molecular dynamics simulations to generate a trajectory are obtained by combining two QM/MM force calculations. A flowchart describing the force calculations is shown in Figure 1. At each time step, the system is partitioned into a number of different regions, which are defined as follows. We begin by creating two sets of atoms, the first consisting of atoms that should follow trajectories using QM forces (we call this the dynamical QM region), and those that should follow MM forces (dynamical MM region). The first and more expensive QM/MM calculation (“extended QM/MM calculation”) uses an enlarged QM region to obtain accurate forces for atoms in the dynamical QM region. This extended QM region is constructed by adding a buffer region around the dynamical QM region. The buffer region size required to reduce the force errors at the dynamical boundary below a preset threshold can be determined from the convergence of forces in the dynamical QM region as a function of buffer region size, carried out separately before the production run on a few relevant configurations (e.g., near the estimated extrema of a free energy profile).

The second QM/MM calculation (“reduced QM/MM calculation”) uses a smaller QM region (which we call the core region) to reduce force errors due to the QM-MM boundary on atoms in the MM region. When the necessary force field parameters are available, the core region may be eliminated altogether and this reduced size QM/MM calculation replaced by a cheap fully MM calculation. We note that the boundary between the dynamical QM and dynamical MM regions does not necessarily need to obey the restrictions that are often put on the QM-MM boundary in a conventional QM/MM calculation, for example, that only single bonds cross the boundary, because it is simply the place at which the source of forces for the dynamics switches. Only the outer boundaries of the core QM region and the buffer region need to obey such restrictions, because those are the boundaries between the QM and MM regions in the two (extended and reduced) QM/MM calculations.

The forces for the propagation of the dynamics are then obtained based on the current identity of the atoms:

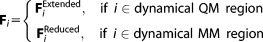
(1)

This is a so-called abrupt force-mixing scheme, where forces used for dynamics switch from one description to the other without a transition region. When an atom is switched from the dynamical QM region to the dynamical MM region or vice versa, the force it experiences has a discontinuity. Introducing a narrow transition region in which the dynamical force is a linear combination of the forces calculated in the extended and reduced QM/MM calculations would smooth out this discontinuity.[12,13,15,17]

**Figure 2:**
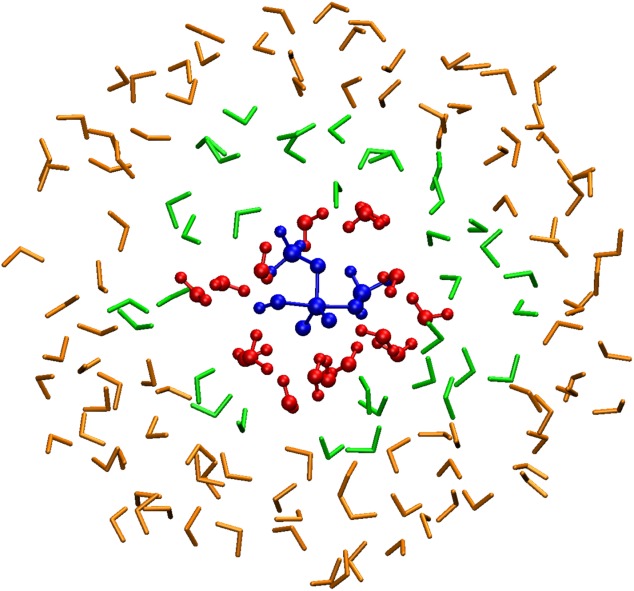
Visualization of the QM regions of an AdBF-QM/MM simulation of dimethyl-phosphate hydrolysis. The core region is the dimethyl-phosphate and the attacking hydroxide ion (blue) with no additional adaptively selected atoms. The dynamical QM region (red) is selected by extending the core region by *r*_qm_ = 3.0–3.5 Å. The buffer region (green) is an additional layer around the dynamical QM region within *r*_buffer_ = 3.0–3.5 Å. The rest of the system (orange) is treated as MM in both the extended and reduced calculations. Ball-and-stick representation is used for atoms which follow QM forces in the dynamics.

Adaptivity is achieved by defining criteria to select atoms for the various regions that are dynamically evaluated at each time step during the simulation. In our implementation, each region is composed of a list of atoms fixed by the user due to their chemical role and additional atoms that are selected due to their distance from atoms in other regions. First, the core region is created by combining the fixed list and nearby atoms, based on a cutoff distance, *r*_core_, from the atoms in the fixed list. Next, the dynamical QM region is defined as the union of the core region, another (optional) fixed list and atoms within a cutoff distance, *r*_qm_, of core region atoms. Finally the buffer region is defined as the union of another optional fixed list and atoms within a cutoff distance, *r*_buffer_, from atoms in the dynamical QM region. An example of these regions from a simulation of the hydrolysis of dimethyl phosphate is shown in Figure 2. To reduce the frequency of switching between regions for atoms that are close to the boundary, hysteresis is applied to all distance cutoffs, so an atom has to come closer than some inner radius to become incorporated into a region, but must move farther than a larger, outer radius to be removed from the region.

The use of force-mixing has two direct consequences stemming from the lack of a total potential energy for the system. First, because the forces are not the derivatives of any energy function, the dynamics are not conservative. The typically very small deviation from linear momentum conservation is easily fixed exactly by adding a correction to some or all forces to ensure that the total force sums to zero, but the deviation from energy conservation necessitates the use of an appropriate thermostat to maintain the correct kinetic temperature throughout the system. We have found that a simple adaptive Langevin thermostat[26] (described below) is sufficient to give a stable and spatially uniform temperature profile.[21] Second, the lack of a total energy prevents the use of some free energy calculation methods, although potential of mean force (PMF) methods, which require only forces and trajectories, can still be applied.[21]

By appropriately setting the cutoff distances for the various regions, the AdBF-QM/MM method can be made to be equivalent to a number of other adaptive methods, summarized in Table1, which we compare to here. The adaptive conventional QM/MM method (AdConv-QM/MM), which is an energy-mixing scheme and is equivalent to the ABRUPT method,[19] corresponds to setting the core and dynamical QM regions to be the same and using an empty buffer region. The adaptive unbuffered force-mixing QM/MM method (AdUF-QM/MM), which is very close to the hot spot method,[12] corresponds to an empty (or minimal) core region, an adaptive dynamical QM region, and an empty buffer region. The difference between the AdConv-QM/MM and AdUF-QM/MM methods lies therefore in how the dynamical forces for the MM atoms are obtained. In the AdConv-QM/MM method there is only one QM/MM force calculation, and the MM atoms are propagated using the forces from this one QM/MM force calculation that gives the forces for the QM atoms. In the AdUF-QM/MM method, which is a true force-mixing approach, the MM atoms are propagated with forces obtained from either a fully MM calculation or a reduced QM/MM calculation with a very small QM region which includes just the reactants. In addition, we also compare our results to a Conv-QM/MM simulation, which is not adaptive, so only the solutes are treated quantum mechanically.

### Implementations of adaptive buffered force-QM/MM method

We have implemented AdBF-QM/MM in two popular QM/MM programs: the AMBER package,[30] which has a number of built in SE methods as well as an interface to external QM programs, and CP2K,[29] which is primarily a DFT package but contains some SE models as well. Because of the different structure of the two codes, the actual implementations are slightly different, so we begin here with the common and general concepts needed to specify an AdBF-QM/MM calculation. In addition to the general QM/MM keywords used by each program the user has to specify only a few additional variables, which are listed in Tables2 and 3.

**Table 2:** New AMBER keywords for AdBF-QM/MM and adaptive thermostats.

Section / Keyword	Explanation
&qmmm	
abfqmmm= 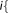 Integer 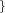	activation of adaptive buffered force QM/MM (*i* = 1)
r_core_in= 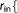 Real 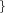	inner hysteretic radius of core region
r_core_out= 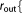 Real 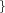	outer hysteretic radius of core region
r_qm_in= 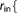 Real 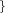	inner hysteretic radius of dynamical QM region
r_qm_out= 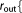 Real 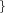	outer hysteretic radius of dynamical QM region
r_buffer_in= 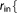 Real 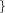	inner hysteretic radius of buffer region
r_buffer_out= 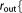 Real 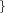	outer hysteretic radius of buffer region
mom_cons_type= 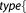 Integer 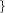	type of momentum conservation
mom_cons_region= 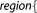 Integer 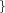	region to apply momentum conservation to
coremask= 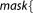 Amber mask 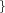	definition of fixed core region
qmmask= 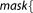 Amber mask 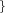	definition of fixed dynamical QM region
buffermask= 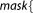 Amber mask 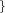	definition of fixed buffer region
corecharge= 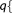 Integer 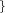	total charge of fixed core region
qmcharge= 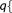 Integer 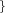	total charge of fixed dynamical QM region
buffercharge= 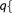 Integer 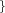	total charge of fixed buffer region
oxidation_number_list_file= 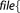 String 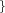	file name containing oxidation numbers
cut_bond_list_file= 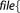 String 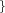	file name containing breakable QM/MM bonds
&cntrl	
ntt= 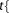 Integer 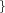	specification of adaptive thermostats (*t* > 4)
gamma_ln= 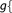 Real 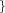	collision frequency for Langevin part of thermostat
nchain= 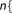 Integer 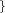	chain length of Nosé–Hoover part of thermostat

Some additional keywords that were used for testing runs are listed in the AMBER manual.

**Table 3:** New CP2K keywords for AdBF-QM/MM and adaptive Langevin thermostats.

Section / Subsection / Keyword	Explanation
&FORCE_EVAL&QMMM	
&FORCE_MIXING	main adaptive QM/MM section
R_CORE 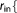 Real 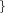 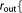 Real 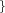	inner and outer hysteretic radii of core region
R_QM 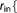 Real 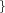 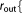 Real 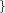	inner and outer hysteretic radii of dynamical QM region
R_BUF 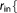 Real 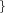 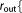 Real 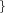	inner and outer hysteretic radii of buffer region
QM_KIND_ELEMENT_MAPPING elem 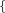 Word 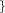 kind 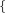 Word 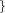	elements to QM kind mapping for adaptively selected atoms
ADAPTIVE_EXCLUDE_MOLECULES mol 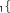 Word 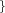 …	list of molecules to exclude from adaptive selection
EXTENDED_DELTA_CHARGE 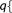 Integer 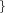	additional net charge in extended region
MAX_N_QM 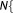 Integer 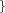	maximum number of atoms allowed in QM region
MOMENTUM_CONSERVATION_TYPE type 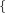 Keyword 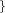	type of momentum conservation
MOMENTUM_CONSERVATION_REGION region 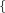 Keyword 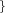	region to apply momentum conservation to
EXTENDED_SEED_IS_ONLY_CORE_LIST 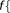 Logical 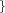	use only core *list* as seed for adaptive dynamical QM region
&QM_NON_ADAPTIVE	definition of fixed dynamical QM region
&QM_KIND kind 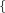 Word 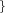	QM kind to use
MM_INDEX 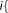 Integer 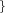 …	list of atoms for fixed dynamical QM region
&END QM_KIND	
&END QM_NON_ADAPTIVE	
&BUF_NON_ADAPTIVE	definition of fixed buffer region
&QM_KIND kind 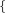 Word 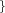	QM kind to use
MM_INDEX 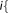 Integer 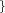 …	list of atoms for fixed buffer region
&END QM_KIND	
&END BUF_NON_ADAPTIVE	
&END FORCE_MIXING	
&MOTION&MD&THERMOSTAT	
TYPE AD_LANGEVIN	type keyword for adaptive Langevin thermostat
&AD_LANGEVIN	
TIMECON_LANGEVIN 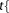 Real 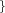	time constant for Langevin part of thermostat
TIMECON_NH 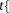 Real 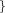	time constant for Nosé–Hoover part of thermostat
&END AD_LANGEVIN	

Possible keyword values are specified in the built-in CP2K documentation. The fixed core region list consists of QM atoms in the enclosing &FORCE_EVAL&QMMM section, whose specification is mandatory in all QM/MM simulations with CP2K.

The most important keywords control the inclusion of atoms in the various regions:

1. Specification of fixed, disjoint lists of core, dynamical QM and buffer atoms. In CP2K the fixed core region cannot be empty; otherwise these lists are optional.
2. Specification of the hysteretic inner (*r*_in_) and outer (*r*_out_) radii of the adaptive core, dynamical QM and buffer regions.

Both the CP2K and AMBER implementations take special care with covalent bonds crossing the boundaries in the reduced and extended QM/MM calculations. To minimize errors associated with breaking such covalent bonds indiscriminately, only entire molecules or fragments bounded by particular covalent bonds are included or excluded from each region. In CP2K, the specific covalent bonds that can be cut by the reduced and extended calculations' interfaces must be fixed in the input file, and large molecules (such as proteins) that should not be entirely included or excluded must therefore be omitted from the adaptive region selection. The AMBER implementation supports an adaptive definition of breakable covalent bonds at the interfaces.

Both implementations support different ways of applying the momentum conservation correction. The CP2K implementation supports different total charges of the QM region in the reduced and extended calculations, as well as constructing the dynamical QM region based only on distances from the fixed subset of the core region. The AMBER implementation automatically adjusts the total charge in the reduced and extended QM/MM calculations based on a default table of oxidation numbers of the adaptively selected atoms. This table can be modified by the user, and the AMBER implementation also supports a number of different geometrical criteria for adaptive core, dynamical QM, and buffer selection.

Adaptive thermostats required for AdBF-QM/MM dynamics have been implemented, including support for independent thermostats for each degree of freedom, using the adaptive Langevin[26] method (CP2K and AMBER) and several variants of the adaptive Nosé–Hoover[26,32,33] method (AMBER only). The keywords used to enable their use are specified in Tables2 and 3. The adaptive Langevin thermostat is essentially a Langevin thermostat (to ensure ergodicity) in parallel with a Nosé–Hoover thermostat (to compensate for deviations from energy conservation), and the corresponding dynamical equations are

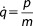
(2)

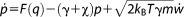
(3)

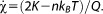
(4)

The position and momentum vectors are *q* and *p*, respectively, *χ* is the Nosé–Hoover degree of freedom, *m* is the atomic mass, and *F*(*q*) is the force. The temperature is *T*, Boltzmann's constant is *k_B_*, *K* is the kinetic energy, and *n* is the number of degrees of freedom associated with the thermostat. The Langevin friction is *γ* = 1*τ_L_* where *τ_L_* is the Langevin time constant, the Nosé–Hoover fictitious mass is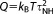 where *τ*_NH_ is the Nosé–Hoover time constant, and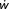 is the time derivative of a Wiener process. The adaptive Nosé–Hoover method has a similar structure, but the Langevin thermostat is replaced with Nosé–Hoover chains with an optional Langevin thermalization of the last thermostat in the chain. In its most general form this gives the adaptive Nosé–Hoover-chains-Langevin method with the corresponding equations

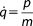
(5)

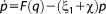
(6)

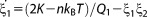
(7)

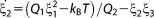
(8)…

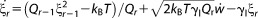
(9)

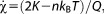
(10)where *r* is the length of the chain, *ξ_i_* and *Q_i_* are the Nosé–Hoover chain degrees of freedom and their masses, respectively, and *γ_l_* is the Langevin friction for thermalizing the final thermostat in the chain. Setting *r* to 1 corresponds to the adaptive Nosé–Hoover-Langevin thermostat, while omitting the Langevin part (i.e., formally setting *γ_l_* to 0) with *r* > 1 results in the adaptive Nosé–Hoover-chain.

Both adaptive thermostats can be applied so that a separate NH variable (or NH chain) is coupled to each degree of freedom, [34] rather than a single NH variable coupling to the total kinetic energy. This is the mode in which we use adaptive thermostats in this work, because in the nonconservative force-mixing simulations extra heat is generated locally near the QM-MM interface and the amount that needs to be dissipated therefore varies in space. We note that a conventional Langevin thermostat operates in a similar way, independently thermalizing of each degree of freedom.

The CP2K inputs consist of a conventional &QMMM section to specify the fixed core list, a &FORCE_MIXING section to specify the other regions and momentum conservation details, and a &THERMOSTAT section with a REGION MASSIVE keyword and an &AD_LANGEVIN section specifying the two time constants. The AMBER input uses new keywords in the &qmmm section to enable force-mixing and set the parameters controlling the various regions, and the ntt keyword with a value of 6, 7, or 8 in the &cntrl section to enable an adaptive thermostat with two new keywords for the Langevin time constant and Nosé–Hoover chain length. Example input files used for some of the simulations presented here are included in the supplementary information. These do not show every available option, and full details are available in the documentation of the two packages.

### Model systems

To test the adaptive QM/MM implementations we studied structure and reaction free energy profiles in three systems. First we validated the new implementations by extending our previous work, which showed that pure bulk water provides a stringent test for adaptive methods,[20] to a number of additional QM models and adaptive QM/MM methods. We then carried out the simulation of two reactions in water solution: the autoprotolysis of water in the presence of a Zn^2^^+^ ion and the hydrolysis of dimethyl-phosphate attacked by a hydroxide ion. The reason we have chosen these reactions was twofold. Both are biologically relevant and widely investigated model reactions for the corresponding enzymatic counterparts. In addition, in both reactions a significant charge transfer occurs between the reactants that require the high level description of the proximate but mobile solvent molecules as well (i.e., at least the first hydration shell). This is especially important in the case of phosphate hydrolysis, which has a highly negative pentavalent intermediate/transition state (TS), making the investigation of this system generally challenging for QM/MM methods. For both reactions, we calculated the free energy profile using a number of adaptive QM/MM methods. In all cases, we compared to reference calculations using a fully QM description using smaller simulation cells, and for the autoprotolysis of water we also ran fully QM simulations using an intermediate size unit cell. The QM region sizes for all QM/MM simulations are summarized in Table4. Adaptive radii were applied to distances between all atoms, except for SE bulk water simulations where only O=O distances were used to select molecules. The sum of core and dynamical QM radii were chosen to ensure that the first hydration shell is included in the dynamical QM region.

**Table 4:** Adaptive region radii for the QM/MM simulations, applied to all interatomic distances, except for SE bulk water simulations (^*^), where the selection criterion was based only on the oxygen–oxygen distances.

Simulation type	*r*_core_ (Å)	*r*_qm_ (Å)	*r*_buffer_ (Å)
SE Bulk water			
AdBF-QM/MM	0.0–0.0	4.0–4.5 (^*^)	4.0–4.5 (^*^)
MNDO/d Autoprotolysis reaction			
Conv-QM/MM	0.0–0.0	0.0–0.0	0.0–0.0
AdConv-QM/MM	2.5–3.0	0.0–0.0	0.0–0.0
AdUF-QM/MM	0.0–0.0	2.5–3.0	0.0–0.0
AdBF-QM/MM	0.0–0.0	2.5–3.0	3.0–3.5
DFT bulk water and dimethyl-phosphate hydrolysis			
Conv-QM/MM	0.0–0.0	0.0–0.0	0.0–0.0
AdConv-QM/MM	3.0–3.5	0.0–0.0	0.0–0.0
AdUF-QM/MM	0.0–0.0	3.0–3.5	0.0–0.0
AdBF-QM/MM	0.0–0.0	3.0–3.5	3.0–3.5

All systems were simulated using constant temperature and volume molecular dynamics. For bulk water, the structure was analyzed by calculating the time averaged radial distribution function (RDF) for a molecule at the center of the dynamical QM region. Free energy profiles were calculated using umbrella integration (UI),[35] with a bias potential

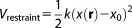
where *k* is the curvature, *x*_0_ is the desired value of the collective coordinate, and *x*(***r***) is its instantaneous value. In the biased simulation the mean gradient of the bias potential is approximately equal to the negative of the gradient of the PMF at the mean value of the collective coordinate.[35] For simulations with AMBER the bias was achieved using the PMFlib package[36] that was linked to AMBER, and for CP2K internal subroutines were used.

#### Bulk water structure

For bulk water we used cubic simulation cells with 13.8 Å (93 molecules) and 41.9 Å (2539 molecules) sides for the fully QM and QM/MM calculations, respectively. The MM water molecules were described with the flexible TIP3P (fTIP3P) potential.[37] We used the AMBER implementation to compare the results of the AdBF-QM/MM method for a number of SE models. In each simulation, a single water molecule was selected to be the center of the dynamical QM region, with radii listed in Table4 applied only to O=O distances when selecting molecules for the adaptive regions. No core region was used, so the reduced size calculation was done as a fully MM calculation. The SE models compared were MNDO,[38] AM1,[39] AM1d,[40] AM1disp,[41] PM3,[42] PM3-MAIS,[43] PM6,[44] RM1,[45] and DFTB.[46] Using the CP2K implementation, we compared the results of various QM/MM methods[47,48] with DFT and the BLYP exchange-correlation functional[49]–[51] plus Grimme's van der Waals correction,[52,53] with a DZVP basis, GTH pseudopotentials,[54] and a density cutoff of 280 Ry. The methods compared were Conv-QM/MM, AdUF-QM/MM, AdConv-QM/MM, and AdBF-QM/MM. In this case a single water molecule was selected for the fixed core region, with adaptive radii listed in Table4 applied to all interatomic distances.

#### Reaction free energy profiles

**Figure 3:**
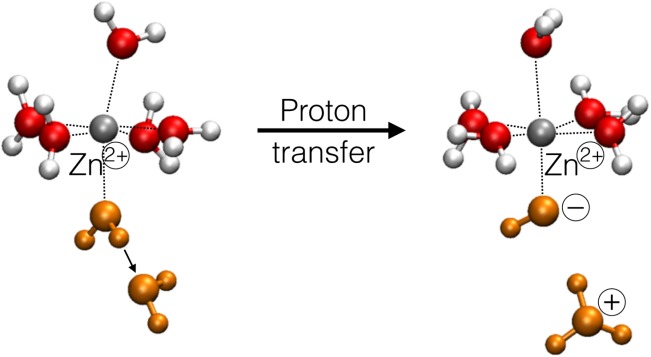
Reaction scheme of the autoprotolysis between a zinc-coordinated and a noncoordinated water molecules (orange). [Color figure can be viewed in the online issue, which is available at http://wileyonlinelibrary.com.]

Water related proton transfer reactions can be facilitated by the presence of divalent metal ions.[55] The metal ion lowers the *p*K*_a_* of the coordinated water molecule making it a stronger acid. Our example is a very simple model of this phenomenon, the proton transfer reaction between a zinc-coordinated water molecule (proton donor) and a noncoordinated water molecule (proton acceptor) in water solution, shown in Figure 3. To calculate the free energy profile for this reaction we used UI with the collective coordinate being the difference between rational coordination numbers (DRCN) of the acceptor and donor oxygen atoms[56,57]:

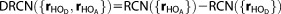
(12)and

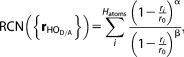
(13)where the subscripts *D* and *A* denote the donor and acceptor oxygen atoms, respectively, *α* = 6, *β* = 18, and the reference distance *r*_0_ = 1.6 Å.

The reactions were simulated in cubic cells with sides of 13.6 Å (87 water molecules) and 17.2 Å (174 water molecules) for the fully QM and 45.8 Å (3303 water molecules) for the QM/MM simulations. The simulations were carried out using the AMBER implementation with Zn^2^^+^ ion parameters from Ref.[58], fTIP3P model for MM waters,[37] and the MNDO/d SE method.[59] The Zn^2^^+^ ion and two reactant water molecules were defined as the QM region in the Conv-QM/MM simulation, as well as the fixed core region in the adaptive simulations. Adaptive region radii are listed in Table4 with all interatomic distances and only entire water molecules were included or excluded in any region.

In all autoprotolysis simulations, we applied one-sided harmonic restraints to the following three distances: between the two O atoms beyond 3.0 Å to keep the reactants together, another between the O atom of donor water molecule and zinc ion beyond 2.5 Å to keep the donor water molecule in the coordination sphere of the metal ion, and the third between the O atom of acceptor water molecule and zinc ion for distances larger than 3.5 Å to prevent the acceptor water molecule from entering into the coordination sphere of the metal ion. For each restraint a force constant of 25.0 kcal mol^–^^1^ Å^−2^ was applied. The applied force constant for the UI restraint was 400 kcal mol^−1^ and the profile was calculated in the range of DRCN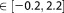.

**Figure 4:**
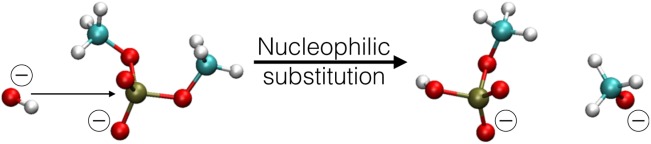
Reaction scheme of the dimethyl-phosphate hydrolysis. [Color figure can be viewed in the online issue, which is available at http://wileyonlinelibrary.com.]

The second reaction we simulated was dimethyl-phosphate hydrolysis, shown in Figure 4, where an incoming hydroxide ion attacks the dimethyl-phosphate and causes a methoxide ion to leave. A similar hydrolysis of phosphate diesters in solution is a biologically important type of phosphoryl transfer reactions and a key model to understand DNA cleavage.[60] The reaction coordinate for the UI procedure was the distance difference between the leaving O=P atoms and the attacking O=P atoms

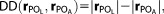
(14)where *L* and *A* designate the leaving and attacking O atoms, respectively. The reaction was simulated in cubic cells with sides of 13.6 Å (86 water molecules) and 48.4 Å (3903 water molecules) for the fully QM and QM/MM simulations, respectively.

Because our simulation protocol starts with an MM relaxation, MM parameters were needed for the solutes. The charges of the phosphate and hydroxide were calculated according to the standard procedure,[61,62] while the bonded and vdW parameters of the phosphate were derived from the ff99SB version of the AMBER force field,[63] and the water molecules were described by the fTIP3P model.[37] For the hydroxide ion the same parameters were used as for the fTIP3P. For the DFT model the BLYP exchange-correlation functional[49]–[51] was applied with Grimme's van der Waals correction,[52,53] using the DZVP basis set with GTH pseudopotentials[54] and a density cutoff of 280 Ry. The QM region of the Conv-QM/MM calculation and the fixed core region of the adaptive QM/MM calculations consisted only of the reactant dimethyl-phosphate and hydroxide. Adaptive region radii are listed in Table4 with all interatomic distances and only entire water molecules were selected for inclusion or exclusion. The free energy profile was carried out in the range of DD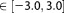 Å using an UI restraint force constant of 400 kcal mol^−1^ Å^−1^.

### Simulation protocol

#### General simulation parameters

All simulations used periodic boundary conditions with MM-MM electrostatic interactions calculated by the Ewald[64] and particle-mesh Ewald[65] for the small and large simulation cells, respectively. For fully QM SE and DFT simulations, the CP2K package was used with the smooth particle mesh Ewald method and multipole expansion up to quadrupoles.[66] In the AMBER QM/MM simulations the QM-MM interactions were calculated using a multipole description within 9 Å while both the long-range QM=QM and QM-MM electrostatic interactions were based on the Mulliken charges of the QM atoms according to Ref.[67]–[70]. In the CP2K QM/MM simulations the QM-MM interaction used Gaussian smearing of the MM charges.[47] When systems were charged a uniform background countercharge was applied. Molecular dynamics simulations with a time step of 0.5 fs were used for equilibration and canonical ensemble sampling.

The first step in the simulation protocol was to generate independent initial configurations for all box sizes from long equilibrium fully MM simulations. In the case of bulk water all fully QM and QM/MM simulations were started from these MM equilibrated configurations. For the reactions, first the relatively computationally inexpensive Conv-QM/MM simulations were carried out starting from an initial configuration that was taken from a fully MM equilibrium simulation at the initial restraint position corresponding to the reactant state. The restraint forces for UI were sampled for some time period, and the restraint center was slowly changed to the next collective coordinate value, then the process repeated until the desired range of values were sampled. The more computationally expensive fully QM and adaptive QM/MM simulations were started from the final configuration of each Conv-QM/MM trajectory at each restraint center position.

#### Initial configurations

The systems and topologies for investigating the bulk water were created by the Leap program of the AMBER package.[30] The initial geometries were relaxed for 5000 minimization steps, followed by a molecular dynamics *NVT* simulation of heating from *T* = 0 K to *T* = 300 K over 50 ps followed by 50 ps at fixed temperature. The density was then relaxed by a 200 ps *NpT* simulation at *T* = 300 K and *p* = 1 bar, and then the average box size was calculated during an additional 500 ps long *NpT* simulation. During this last stage 10 independent configurations were selected at 50 ps intervals, which were all rescaled to the mean volume. Finally, for each of the 10 configurations a 500 ps long *NVT* simulation was carried out at 300 K. In each case the temperature was controlled by a Langevin thermostat[71] with a friction coefficient of 5 ps^−1^. The systems for the reactions were also generated using the Leap program of the AMBER package[30] to surround the reactants by water molecules. These starting configurations were equilibrated by the same procedure as for the bulk water systems.

#### Water autoprotolysis

Conventional QM/MM simulations were carried out using AMBER and PMFlib for DRCN from −0.2 to 2.2 in increments of 0.1. The restraint reaction coordinate was changed from its actual value in the reactant state to the starting value of −0.2 over 20 ps. Then, the DRCN was sequentially changed by 0.1 over 1 ps long trajectories, followed by simulation at fixed restraint position. Restraint force values for UI were collected for the number of initial configurations and trajectory lengths listed in Table5. All simulations used a Langevin thermostat[71] with a friction coefficient of 5 ps^−1^.

**Table 5:** Configuration numbers and trajectory lengths.

Simulation type	# of independent configurations	Trajectory length per config.	
		total (ps)	used for analysis (ps)
Bulk water			
SE fully QM	10	10	5
SE AdBF-QM/MM	10	50	40
DFT fully QM	5	10	9
Autoprotolysis reaction			
MNDO/d fully QM	10	12	10
MNDO/d Conv-QM/MM	10	10	8
MNDO/d AdConv-QM/MM	10	5.5	4.5
MNDO/d AdUF-QM/MM	10	5.5	4.5
MNDO/d AdBF-QM/MM	10	5.5	4.5
Dimethyl-phosphate hydrolysis reaction			
DFT fully QM	5	5	2.5

Fully QM simulations for both box sizes were carried out using CP2K, starting from relaxed Conv-QM/MM configurations at each reaction coordinate value, with a number of independent initial configurations and trajectory lengths listed in Table5. Temperature was controlled by the CSVR thermostat[72] with a time constant of 200 fs. Adaptive QM/MM simulations were carried out starting from relaxed Conv-QM/MM for the number of initial configurations and trajectory lengths listed in Table5. Because of the energy conservation violation of all the adaptive methods, temperature was controlled by adaptive Langevin thermostats,[26] one per degree of freedom, with a Langevin time constant of 200 fs and a Nosé–Hoover time constant of 200 fs.

#### Dimethyl-phosphate hydrolysis

Initial conditions for the DFT simulations were generated by a Conv-QM/MM simulation with the AM1 SE method using the AMBER code for DD from −3.0 to 3.0 Å. The DD was changed from its initial value to −2.0 Å over 20 ps. The DD was then changed by increments of 0.1 Å over 1 ps, followed by equilibration for 10 ps at each DD value. All subsequent simulations were carried out using CP2K using one adaptive Langevin thermostat per degree of freedom with a Langevin time constant of 300 fs and a Nosé–Hoover time constant of 74 fs. Simulations with fully QM, Conv-QM/MM, AdConv-QM/MM, AdUF-QM/MM, and AdBF-QM/MM were carried out with the number of configurations and trajectory lengths listed in Table5. Values of DD from −3.0 Å in increments of 0.6 Å, with additional samples at DD = ±0.3 Å and DD = ±0.1 Å, were used to calculate the UI free energy profile.

## Results

### Bulk water

**Figure 5:**
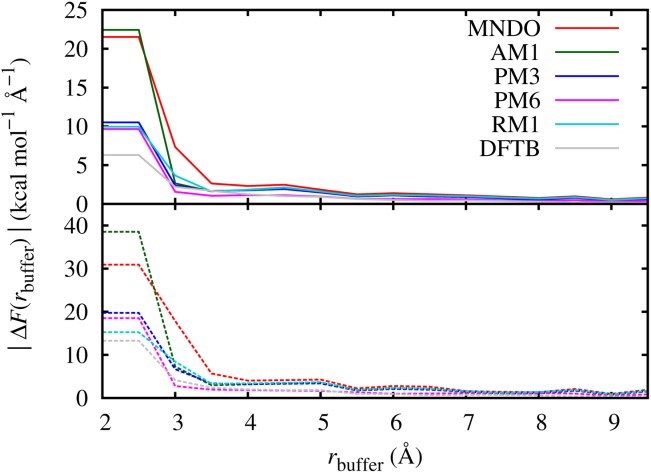
Force convergence on the central oxygen atom in pure bulk water for different SE methods relative to reference forces from calculations using the same SE method with buffer size of 10.0 Å. Top panel shows the mean force error based on 10 independent configurations, and bottom panel shows the maximum error. [Color figure can be viewed in the online issue, which is available at http://wileyonlinelibrary.com.]

We performed a force convergence test to determine the appropriate buffer radii by calculating the forces on an O atom in the center of the QM region of a conventional QM/MM calculation, as a function of QM region radius, using a number of SE methods. Here the radius of the QM region is equivalent to *r*_buffer_ in the AdBF-QM/MM method's extended QM calculation, as it controls the distance between the molecules whose forces we are testing and the QM-MM interface. The atomic configurations were taken from the 10 MM equilibrated configurations described in the Simulation Protocol subsection and the calculations were carried out with MNDO, AM1, PM3, PM6, RM1, and DFTB. The resulting force errors calculated with respect to reference forces from a 10 Å radius conventional QM/MM calculation are plotted in Figure 5. For each QM method a similar behavior is seen in the force convergence: the average force error goes below 2 kcal mol^−1^ Å^−1^ (and the maximum goes below 4 kcal mol^−1^ Å^−1^) around *r*_buffer_ = 4.0 Å, which was chosen as the lower limit of the buffer size for the dynamics. A similar behavior was observed in the case of DFT (BLYP).[20] We also investigated forces on the hydrogen atoms (data not shown) and found a slightly faster convergence, reaching the same average force error with *r*_buffer_ that is 0.5 Å smaller.

**Figure 6:**
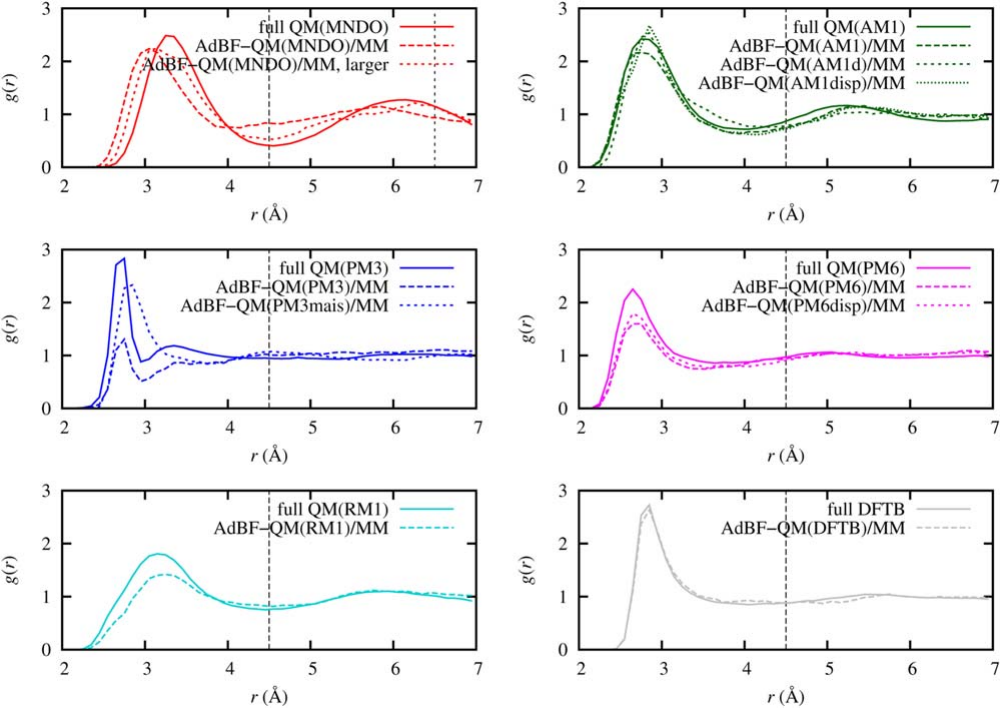
Oxygen–oxygen RDFs in bulk water using different SE methods. Vertical dashed lines at 4.5 Å denote the size of dynamical QM region. For MNDO, a second vertical line at 6.5 Å represents the outer boundary of the dynamical QM region for the larger simulation. [Color figure can be viewed in the online issue, which is available at http://wileyonlinelibrary.com.]

The oxygen–oxygen RDFs averaged over 10 independent trajectories are plotted in Figure 6. In the case of PM3 the fluid density gradually goes down in the dynamical QM region during the dynamics and longer simulations showed that this process is irreversible, leading to an almost complete depletion of water in the dynamical QM region. This phenomenon was observed previously[73] and the significantly different diffusion constants of the QM and MM water models were suggested as a possible reason. The PM3-MAIS method, which is an extension to PM3 parametrized to accurately reproduce the intermolecular interaction potential of water, does not suffer from this problem. In contrast to PM3, in case of MNDO the water structure in the QM region is stable for the duration of our simulations but the RDF slightly differs from the fully QM result. As expected, using a larger QM region improves the structure in this case. We also note that the force convergence for the MNDO is the slowest among the examined potentials (Fig. 5), so a larger buffer region may further improve the RDF. In the case of PM6 and RM1, the AdBF-QM/MM RDFs show a somewhat lower first peak compared to the fully QM structure. However, the RDFs remain stable for longer simulation times. Based on our data we are not able to exclude unambiguously the possibility that, similarly to PM3, a net flux of atoms leaving the dynamical QM region causes this discrepancy. Even if this is the case, the diffusion is much slower than for PM3. For DFTB and AM1 the AdBF-QM/MM and fully QM RDFs match well. We investigated two additional AM1 variants (AM1d and AM1disp) and found similar RDFs to the fully QM AM1 result. In general we see that the first peak is higher for the fully QM simulations than those of AdBF-QM/MM. Although using larger dynamical QM and buffer regions could potentially improve the agreement, the improvement may be limited by differences in how the long range interactions are calculated[67,69,74] in the fully QM and the AdBF-QM/MM simulations due to limitations in the packages used (CP2K and AMBER, respectively).

To show the computational cost of the various adaptive QM/MM methods, in Table6 we list the average computational time (wall time multiplied by number of cores), number of electrons in QM region, and QM calculation cell size for each method from the CP2K pure water DFT simulations. The times and sizes are averaged over the first 500 steps, before the unphysical ejection of most water molecules from the adaptive region in the AdUF-QM/MM method. The cell sizes were chosen to accomodate the maximum possible size of the extended QM calculation, based on the core region (one molecule) and hysteretic radii. The runs were performed on an SGI ICE X with dual 8-core 2.6 GHz Intel E5 CPUs and FDR Infiniband interconnect. Note that for AdConv-QM/MM we list half the actual runtime, because as implemented in CP2K the software carries out two identical calculations, unfairly increasing the cost of the method, which could trivially be optimized by only carrying out one. The time for the AdUF-QM/MM calculation is about double that of AdConv-QM/MM because, as implemented in CP2K, the AdUF-QM/MM method requires two QM/MM calculations, which have nearly equal cost because they use the same QM cell size despite the different numbers of atoms in the reduced and extended calculations.

**Table 6:** System size and computation time parameters for CP2K DFT bulk water simulations averaged over the first 500 steps.

Method	Comp. time per step (s)	# of electrons (extended/reduced)	QM cell size (Å)
Conv-QM/MM	27.2	8	9.5
AdConv-QM/MM	188.8	80	20.0
AdUF-QM/MM	361.6	72 / 8	20.0
AdBF-QM/MM	1398.4	312 / 8	30.0

Computational time per MD step is summed over parallel processes, and numbers of electrons for both extended and reduced QM region calculations are listed (the same cell size used for both). Note that actual runtime for AdConv-QM/MM method has been halved, as discussed in the text.

**Figure 7:**
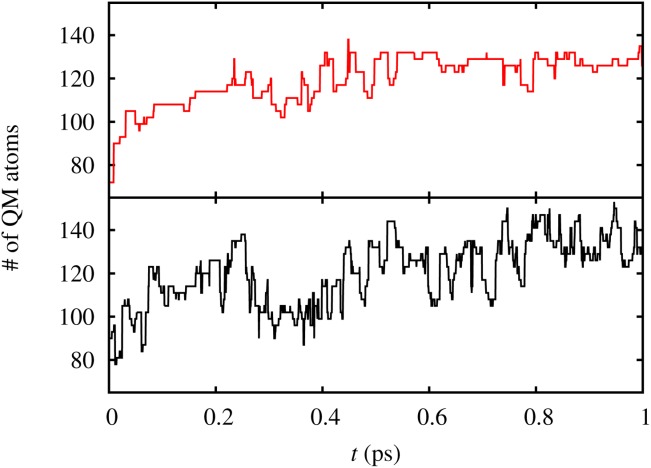
Number of QM atoms in the extended calculation from the AdBF-QM/MM CP2K DFT bulk water simulation with (top graph) and without (bottom graph) hysteresis. [Color figure can be viewed in the online issue, which is available at http://wileyonlinelibrary.com.]

Figure 7 shows the total number of QM atoms in the extended calculation of the AdBF-QM/MM method with and without using hysteresis in the definition of the adaptive regions for a portion of the trajectory. For the nonhysteretic case we used the same trajectory obtained from a simulation with radii shown in Table4 and recalculated the number of QM atoms using the coresponding average values for the inner and outer radii (i.e., 3.25 Å for all dynamical QM and buffer radii). Using hysteretic radii considerably reduces the fluctuation in the number of QM atoms, in line with previously work.[20]

**Figure 8:**
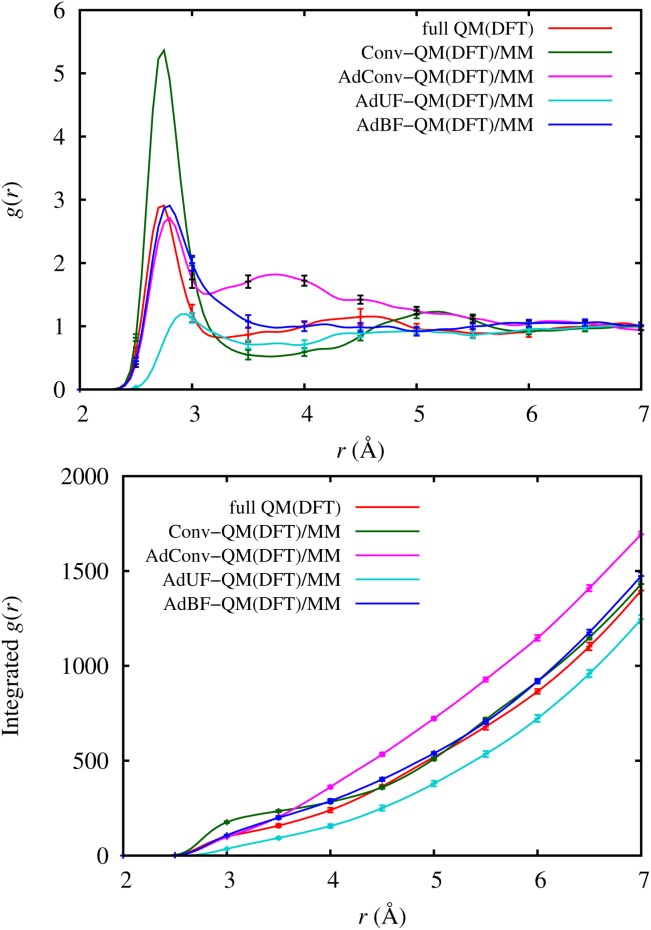
RDFs and integrated RDFs of bulk water using DFT with different adaptive QM/MM methods. [Color figure can be viewed in the online issue, which is available at http://wileyonlinelibrary.com.]

In Figure 8, we compare the O=O RDFs computed using DFT and Conv-QM/MM, AdConv-QM/MM, AdUF-QM/MM, AdBF-QM/MM, and fully QM methods. All but AdUF-QM/MM have a first neighbor peak at approximately the correct distance, but their heights vary greatly. In the Conv-QM/MM calculation, where only a single water molecule is in the QM region, the first neighbor peak height is approximately double the fully QM value, indicating that inaccurate forces at the QM-MM interface are greatly distorting the structure around the QM water. In the AdConv-QM/MM calculation, where the size of the dynamical QM region is increased using hysteretic radii of 3.0–3.5 Å, the peak height is greatly improved, but there is an excess of molecules just inside the QM-MM interface, leading to an unphysical second broad peak in the RDF centered around about 3.8 Å. In contrast, using force mixing without buffers in the AdUF-QM/MM calculation leads to an emptying of the dynamical QM region, nearly completely eliminating the first neighbor peak. The AdBF-QM/MM method comes closest to reproducing the fully QM structure. The first neighbor peak has the correct position and height, although the minimum near 3.2 Å has been replaced by a shoulder. This artifact may be caused by the nearby QM-MM interface, and could perhaps be corrected by a larger dynamical QM region. Note that the effect is already much less significant than the artifacts in the other adaptive methods. The cumulative RDFs in the bottom panel of Figure 8 show corresponding differences between the methods. The Conv-QM/MM curve shows a large bulge near the first peak, but then follows the fully QM curve at longer distances due to an overly deep minimum in the RDF that compensates for the excess first neighbors. The two unbuffered adaptive methods show significant deviations from fully QM, up for AdConv-QM/MM which has an excess second RDF peak, and down for AdUF-QM/MM which is missing the first peak. Our AdBF-QM/MM results show better agreement with fully QM throughout the distance range, with a small offset to larger values starting after the first RDF peak due to the shoulder in the peak.

### Water autoprotolysis in the presence of a zinc ion

In the simulation of water autoprotolysis in the presence of a Zn^2^^+^ ion with the Conv-QM/MM method, the QM region consists of the metal ion and the reactant water molecules. No additional water molecules from the zinc's coordination sphere are included because they are mobile so can exchange with bulk phase water on the simulation time scale, and the Conv-QM/MM method is not adaptive. A possible way to keep additional water molecules in the dynamical QM region would be to restrain them near the zinc ion,[75] or restrain the remaining waters away from the dynamical QM region.[76] However, such restraints can significantly affect the entropic part of the free energy.[57]

**Figure 9:**
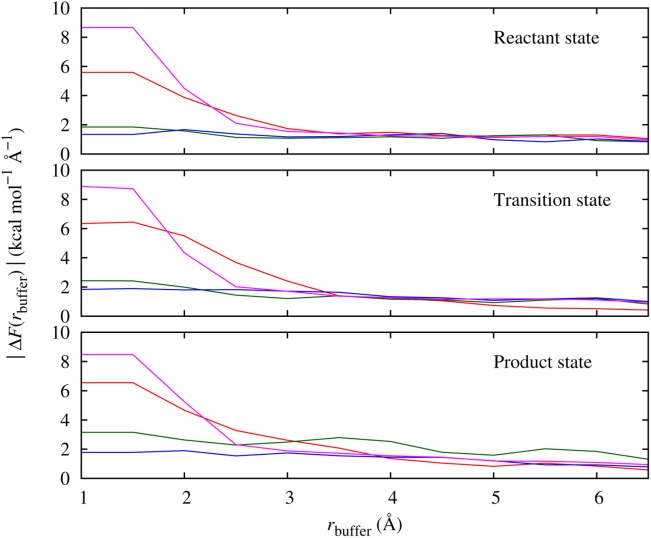
Mean force errors of key atoms in the dynamical QM region (*r*_qm_ = 3.0 Å) of the water autoprotolysis reaction using the MNDO/d model and different sizes of buffer region at the three Conv-QM/MM predicted extremum points, relative to forces from a calculation with buffer size of 7.0 Å. Force errors on zinc ion (red), donor (green) and acceptor (blue) oxygen atoms and the average of nonreactive oxygen atoms (purple) in the dynamical QM region are shown. [Color figure can be viewed in the online issue, which is available at http://wileyonlinelibrary.com.]

For the adaptive QM/MM simulations, we found that *r*_qm_ = 2.5–3.0 Å was sufficient to include the first hydration shell around the zinc ion and the reactants. To obtain the values of *r*_buffer_ we carried out force convergence tests at geometries taken from the free energy profile extremum states (reactant, transition and product) from the Conv-QM/MM simulation. The average and maximum force errors of the zinc, the donor and acceptor oxygen atoms (which together comprise the core region) and the oxygen atoms of nonreacting water molecules in the dynamical QM region are plotted in Figure 9. We see that including the first hydration shell around the reactant water molecules is sufficient to reduce the force error on all atoms to below approximately 2.5 kcal mol^−1^ Å^−1^, which we take to be an acceptable value. Similarly to bulk water, the hydrogen atoms have a slightly faster convergence reaching the same average force error with *r*_buffer_ that is 1.0–1.5 Å smaller (data not shown). Interestingly, force errors on the metal ion require *r*_buffer_ ≥ 3.0 Å to reach equally small values, despite the fact that it is surrounded by QM waters in its first coordination sphere even without the use of a buffer region. The reason for this slow convergence is probably due to the metal ion's high charge and polarizability, which cannot be fully screened by the coordinated water molecules. Based on the convergence of the force on metal ion and the nonreactive water molecules in the dynamical QM region we chose *r*_buffer_ = 3.0–3.5 Å. As the force convergence test showed small errors on the reactants' atoms (although not on the metal ion, which functions as a catalyst, not a reactant) even in the absence of any buffer region, it may be reasonable to carry out the simulations without a buffer. We therefore also performed AdConv-QM/MM and AdUF-QM/MM simulations using our AMBER implementation.

**Figure 10:**
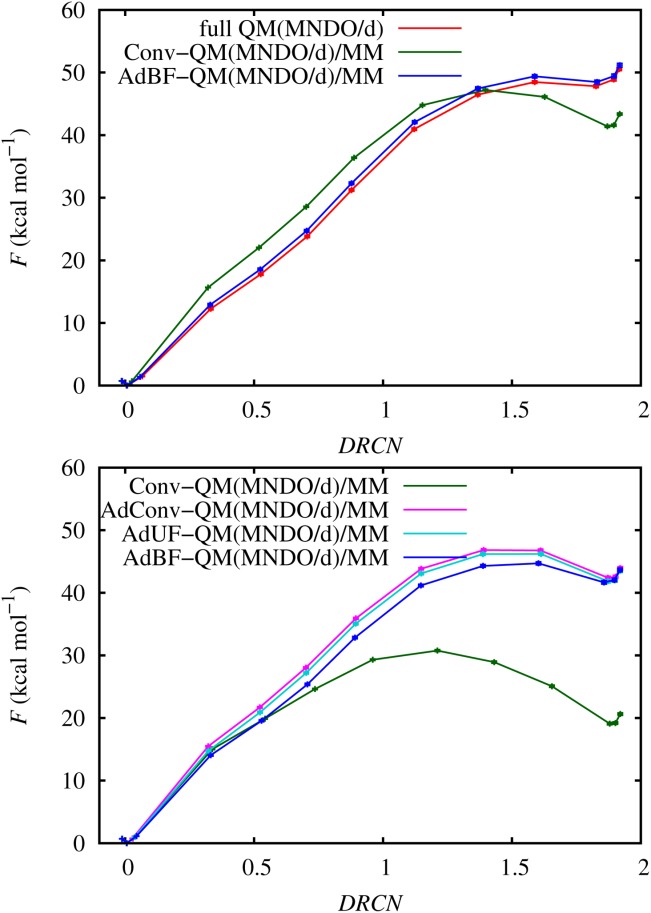
PMF profiles of the water autoprolysis reaction using MNDO/d and the different QM/MM methods as functions of the difference of rational coordination number DRCN. 95% confidence intervals are comparable in size to symbols. Top panel shows results from CP2K including periodic fully QM SE simulation, and bottom panel shows results from AMBER. [Color figure can be viewed in the online issue, which is available at http://wileyonlinelibrary.com.]

The free energy profiles of the different adaptive QM/MM methods calculated with the CP2K and AMBER implementations are presented in Figure 10. As the formulations of the QM-MM interaction and the way periodic boundary conditions are applied differ for the two programs, they are not directly comparable, so we show the corresponding profiles in different panels. For all cases DRCN = 0.0 corresponds to the reactant state. We used the profile of the smaller fully QM unit cell size as reference, but as the larger fully QM unit cell size profile differs by less than 0.025 kcal mol^−1^ RMS, we conclude that the small QM unit cell profile is converged with respect to the unit cell size. The curve of the fully QM simulation shows the TS at around DRCN = 1.6 with an activation barrier of 48.5 kcal mol^−1^ and a shallow minimum of the product state at DRCN ∼ 1.8 with a reaction free energy of 47.8 kcal mol^−1^.

As the reaction proceeds the Conv-QM/MM profile diverges from the rest. However, the deviation is much larger for the AMBER implementation than for CP2K. This is probably due to the differences in calculating the QM-MM interaction in the two programs; for example, the replacement of the point charges used in AMBER by Gaussians in CP2K may be reducing the overpolarization of the QM calculation by the MM region and leading to an improvement of the Conv-QM/MM calculation. In CP2K, the adaptive method reproduces the fully QM result, in contrast to Conv-QM/MM. In AMBER, the AdConv-QM/MM and AdUF-QM/MM profiles differ only slightly from the AdBF-QM/MM one, but all differ greatly from the Conv-QM/MM result. This difference is due to the larger QM region in the adaptive simulations, in accord with the observation of the force convergence test where a QM region that included the first hydration shell was sufficient to get force convergence on the atoms of the reactants, even without the use of a buffer region. The overall difference between the CP2K and AMBER results is presumably due to their differing treatment of periodic boundaries on the QM region, as mentioned above.

### Dimethyl-phosphate hydrolysis

To determine the sizes of the QM and buffer regions, we carried out force convergence tests of the three key atoms of the system that are involved in the reaction coordinate DD: the phosphorus atom and the attacking and leaving oxygen atoms. First, we examined the effect of different buffer region sizes directly around the phosphate and hydroxide ions by varying *r*_buffer_ with *r*_qm_ = 0.0 Å (Fig. 1). Because a full QM reference calculation is not possible, we compared our forces to a QM/MM calculation with the largest QM region feasible, *r*_buffer_ = 7 Å.

**Figure 11:**
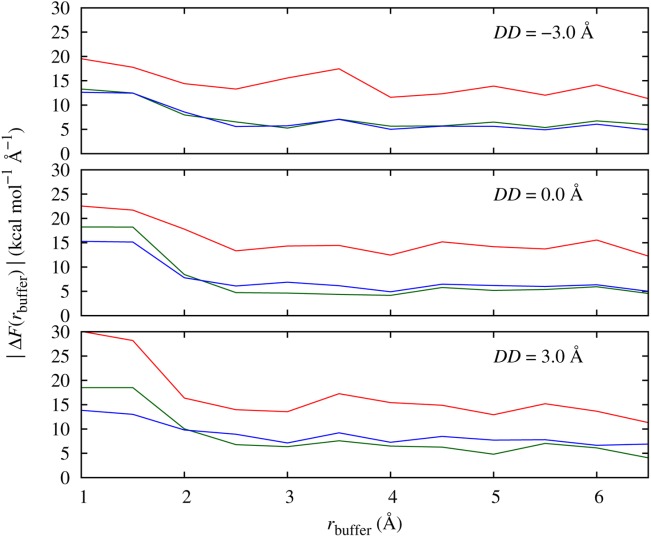
Mean force errors of key atoms of the phosphate hydrolysis reaction using DFT and different sizes of buffer region around the phosphate – hydroxide ion system (i.e., *r*_qm_ = 0.0 Å) at three different DD values, relative to reference forces from a calculation with buffer size of 7.0 Å. Force errors on phosphorus atom (red), attacking (green) and leaving (blue) oxygen atoms are shown. [Color figure can be viewed in the online issue, which is available at http://wileyonlinelibrary.com.]

For the oxygen atoms, a similar behavior of the profiles can be seen for all three DD values we investigated: without a buffer (i.e., *r*_buffer_ = 1.0 Å, which is too small to include any neighboring molecules) the error is about 15–20 kcal mol^−1^ Å^−1^, and it goes down to 5–6 kcal mol^−1^ Å^−1^ using a buffer size of 3.0–3.5 Å. This buffer size corresponds to the first hydration shell around the reactants, and applying a larger buffer size does not improve the force convergence. For the phosphorus atom, the force convergence profile shows a similar behavior but converges to a larger average force error of ∼15 kcal mol^−1^ Å^−1^. The observed lack of systematic convergence of the forces to those of our reference QM/MM calculation indicates either that the QM region size we used for the reference is far from the converged fully QM calculation limit, or that the Conv-QM/MM method does not actually converge with QM region size to the fully QM result. Either of these possibilities reflects the limitations, in this system at least, of the mechanical and electrostatic boundary conditions in the conventional QM/MM method used. Based on Figure 1 we set *r*_qm_ = 3.0–3.5 Å. We also investigated the convergence of forces as a function of buffer region around a finite dynamical QM region (*r*_qm_ = 3.0–3.5 Å). In this case, we did not find any additional improvement of the force convergence, which is in agreement with the tail of the profiles in Figure 1 and suggests that applying a buffer region beyond the dynamical QM region that includes the first hydration shell will not alter the free energy profile significantly. We tested this using *r*_buffer_ = 3.0–3.5 Å, as in the other simulated systems, in the AdBF-QM/MM calculations.

The free energy curves of the conventional and adaptive QM/MM simulations of the systems are shown in Figure 2. All profiles are shifted to *F* = 0 kcal mol^−1^ at DD = –3.0 Å. The fully QM profile has a maximum at DD = –0.3 Å with Δ*F*^‡^ = 22.0 kcal mol^−1^, indicating the TS of the reaction. Within the range of the UI calculations ([–3.0, 3.0"left" Å) the fully QM profile does not have minima as expected due to the repulsion of the negatively charged reactants and products. The Conv-QM/MM simulations result in a wide flat region in the range [–0.6, 0.6"left" Å with a minimum at DD = 0.0 Å, indicating a possible intermediate metastable state rather than a TS, although the observed minimum is shallower than the error bars. The top of the Conv-QM/MM profile is lower by ∼5 kcal mol^−1^ than the peak of the fully QM curve, corresponding to an error of ∼25%. The AdUF-QM/MM profile has a single well defined TS, but its height is significantly overestimated compared to fully QM (Δ*F*^‡^ = 32.8 kcal mol^−1^). In contrast to Conv-QM/MM and AdUF-QM/MM, both the AdConv-QM/MM and AdBF-QM/MM profiles are in good agreement with the fully QM profile.

**Figure 12:**
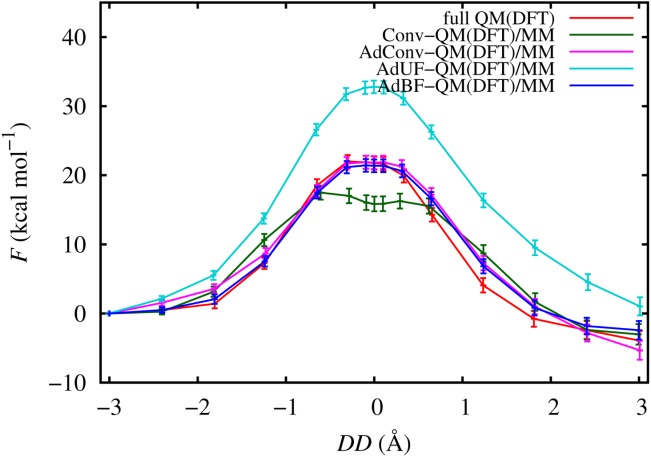
PMF profiles of the phosphate hydrolysis reaction using DFT and the different adaptive QM/MM methods as functions of the distance difference *DD*, with 95% confidence intervals. [Color figure can be viewed in the online issue, which is available at http://wileyonlinelibrary.com.]

To further investigate the source of these differences, we computed the RDFs between the central phosphorous and all water oxygen atoms. Instead of using the RDF, which is noisy due to the relatively short simulation times, we calculated its integral (IRDF), shown in Figure 3. For smaller distances (2.5–3.0 Å) the Conv-QM/MM IRDF shows a higher water density around the reactants compared to the fully QM simulations. This is due to the ability of the MM hydrogen atoms to approach the pentavalent TS too closely, leading to an overstabilization of the doubly negatively charged phosphate and resulting in a lower barrier. In the case of AdUF-QM/MM the IRDF profile shows that an instability has pushed water molecules out of the dynamical QM region, decreasing the density for *r* at least up to 7 Å. This unphysically low density in the reaction region reduces the stabilization of the TS by the nearby waters, in accord with the higher barrier observed. In the vicinity of the reactants, both the AdConv-QM/MM and AdBF-QM/MM integrated RDFs are close to the fully QM one. At larger distances (starting from 4 Å), the AdConv-QM/MM RDF starts to diverge while AdBF-QM/MM remains closer to the fully QM result, although the AdConv-QM/MM method's structural error does not significantly affect its free energy profile.

**Figure 13:**
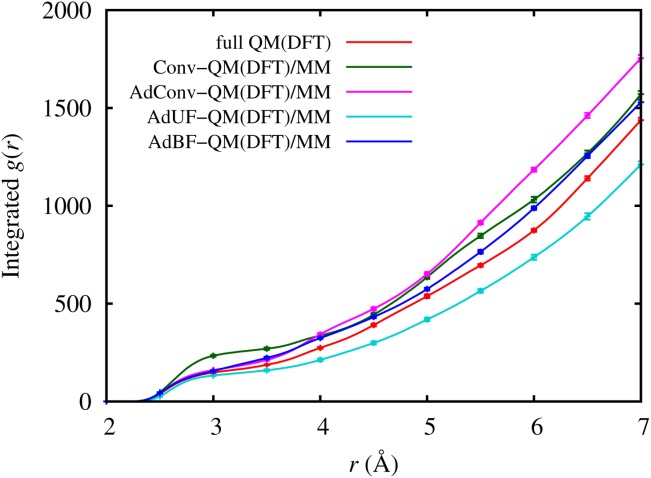
Integrated central phosphorus–water oxygen RDF at the TS corresponding to the fully QM simulation of the phosphate hydrolysis reactions using different adaptive QM/MM methods, with 95% confidence intervals. [Color figure can be viewed in the online issue, which is available at http://wileyonlinelibrary.com.]

## Discussion and Conclusions

The QM/MM approach has been widely used for simulating processes that require a quantum-mechanical description in a small region, for example, a reaction with covalent bond rearrangement, within a larger system with important long-range structure, such as a protein or a polar solvent. However, conventional approaches are limited to a fixed QM region, and also contain significant errors in atomic forces near the QM-MM interface as compared with fully QM or fully MM simulations. Making the QM region larger can help by moving the QM-MM interface further away from the region of interest, but may require the methods to become adaptive and allow molecules to diffuse into or out of the QM region. Such adaptive methods have been developed, but it has proven difficult to make them stable, at least partly because the force errors near the QM-MM interface can unphysically drive particles from one region to the other. To address these issues and enable stable adaptive simulations we have developed the AdBF-QM/MM method, which reduces interface errors by combining forces from two QM/MM calculations with different QM sizes using force-mixing. Here we have described its new implementation in the CP2K and AMBER programs, building on their existing QM/MM capabilities. Using the new functionality requires the specification of a few parameters to control the sizes of the core QM, dynamical QM, and buffer regions. The AdBF-QM/MM method and its implementations are formulated in a general way, so they can be used with a wide range of QM and MM models as well as different treatments of the QM/MM boundary when performing the force calculation.

We have tested our implementations using a variety of QM models, including both SE and DFT, on several structural and free energy problems, using conventional QM/MM, AdBF-QM/MM, as well as other adaptive methods that forgo the use of some of the QM and/or MM buffer regions. Using the CP2K and AMBER implementations we simulated the structure of bulk water, which was previously used as a stringent test of an earlier implementation of the AdBF-QM/MM method,[20] and shown that the new implementations of AdBF-QM/MM produce a stable structure in good agreement with fully QM simulations for DFT and for some, but not all, SE methods we tested. We also performed two new tests of adaptive QM/MM methods, a comparison of the free energy profiles of two reactions, water autoprotolysis in the presence of a Zn^2^^+^ ion (SE using AMBER) and dimethyl-phosphate hydrolysis (DFT using CP2K), to fully QM results. We found that the profiles show a substantial dependence on the choice of adaptivity, buffers, and details of the QM-MM interaction term. In all cases, the use of a simulation that includes at least one hydration shell beyond the reacting species is important for reproducing the fully QM free energy profile. The water autoprotolysis simulations show that all the adaptive methods, which include a hydration shell in the QM region, are in agreement with each other, except for some differences between AMBER and CP2K due to their differing QM-MM interactions. Where such a comparison can be made they also agree with a fully QM calculation, and differ significantly from a Conv-QM/MM calculation that does not include the hydration shell. The dimethyl phosphate hydrolysis simulations show that the free energy profiles of the AdConv-QM/MM and AdBF-QM/MM adaptive method are in good agreement with fully QM results, while the Conv-QM/MM and AdUF-QM/MM methods are not. The reason for this difference is that the former two methods result in a reasonable solvent structure around the reaction, while the latter two give very different structures. The Conv-QM/MM simulation also predicts a qualitatively incorrect metastable state at the TS collective coordinate value.

In summary, our results show that of the adaptive methods we have tested, the AdBF-QM/MM method is the most robust in maintaining reasonable solvent structure and giving accurate free energy profiles, although using the buffer incurs a significant computational cost. Adaptive methods that do not include both dynamical QM and buffer regions can also give good structural and free energy profile results for some systems, but they fail to agree with fully QM results for other systems. To maximize the accuracy of the AdBF-QM/MM method the size of core region should be minimized, the dynamical QM region should include at least one hydration shell around the reaction centre so as to include the most important solvent effects, and the buffer region should be large enough to give forces throughout the dynamical QM region that are converged to better than a few kcal mol^−1^ Å^−1^. Our AMBER and CP2K implementations use a small number of simple parameters to specify the various adaptive regions, and the suggested size criteria can be satisfied with reasonable computational cost, making the AdBF-QM/MM method accessible to a wide community of users.
